# Reading: From the Simple to the Complex

**DOI:** 10.3390/brainsci12121670

**Published:** 2022-12-05

**Authors:** Hermundur Sigmundsson, Helga S. Thórsdóttir, Herdís R. Njálsdóttir, Svava Th. Hjaltalín

**Affiliations:** 1 Department of Psychology, Norwegian University of Science and Technology, 7491 Trondheim, Norway; 2 Research Center for Education and Mindset, University of Iceland, 101 Reykjavik, Iceland

**Keywords:** children, letter-sound knowledge, READ, breaking the reading code, reading, challenges, learning, plasticity

## Abstract

The aim of this article was to present an important perspective on reading skill development. The perspective ‘READ’ builds on the phonics approach which has been found to be most important in relation to reading achievement i.e., to teach children to break the reading code. In addition, READ builds on theories within learning and skill development. The Ericsson concept of ‘deliberate practice’ refer to baseline measurements that provide a basis for follow-up and deliberate practice. The concept of ‘flow’ is also of great importance where challenges are always in relation to the skills. It means that each child will be able to experience ‘flow’ where mastery is the key word, feeling I CAN! When mastery is experienced, the dopamine hormone gives the feeling of reward. Stimuli, experience, and repetition is also a key word in the ‘training hour’ where children get the possibility to strengthen the neural network that is used for specific skills which are trained. In this respect, the letter-sound knowledge is trained until the child has broken the reading code. The results from the first year in the school in Vestmannaeyjar in Iceland indicates that all the children were able to break the reading code or read simple words. In addition, 96% of the children were able to read sentences, and 88% where able to read text. These promising results are discussed in relation to Ericsson’s and Csikszentmihalyi’s important theories.

## 1. Introduction

The ‘Ignition’ approach (‘Kveikjum neistann’), that is now being followed in the first grade at Vestmanna Islands Elementary School, is about placing great emphasis on reading instruction for beginners. The goal is for 80–90% of students to be considered fully literate by the end of second grade. A status assessment (Letter-sound knowledge test, LSK test, [1,2,3]) is used from the first month in schools, which ensures compliance and ensures that each child’s challenge is in accordance with their skills [4,5]. With a position assessment (evaluation) in the basic aspects of reading at the beginning of school, teachers can monitor the status of students and provide them with strong support right from the start of their reading studies. Children come differently prepared for their study, but all have their strengths and weaknesses [6]. It is therefore very important to map their position right at the beginning of the reading program and provide them with appropriate training and challenges, what Ericsson calls ‘deliberate practice’ [7]. Ericsson’s theory emphasizes the importance of systematic training over time with well-defined specific goals. Deliberate practice requires the child to constantly try things that are just beyond his or her current skills. It is deliberate and requires the child’s «full attention and conscious actions» [6] (p. 99). That perspective is supported by research within neuroscience, i.e., brain plasticity. That is experience-dependent, which may reflect experience and input from the environment. This input may vary a lot between different individuals and supports learning throughout life [8]. For example, Edelman [9,10], with his theory on ‘neural Darwinism’, pointed out that for skill to be automatized it takes lot of repetition. The systematic training and repetition create and develop networks in the brain. With more specific training, the stronger the network will become. That may be the general principle for all skills and knowledge development, i.e., what is trained develops! [11,12]. Research has also indicated that «the efficacy of synaptic signaling in the PFC (pre-frontal cortex network) is profoundly influenced by monoaminergic inputs via the activation of dopamine, adrenergic, or serotonin receptors» [13] (p. 445).

Vestmanna Island is a group of 14 small Icelandic islands off Iceland’s southern shore. Heimaey (4 miles, 6 km in length) is the largest and only inhabited island in which the town Vestmannaeyjar is located. Fishing and tourism are the chief economic activities. The population of the islands is around 4.000. The entire sample of children in the first grade reflected the population of children attending schools in the region and included children in a wide range of socio-economic backgrounds.

## 2. The Simple View of Reading

Phonics adapts the simple view of reading [14]. The simple view of reading [15] has attracted a lot of attention in the last 30 years. Accordingly, reading consists of two components: decoding and language comprehension. It is therefore not possible to talk about active reading unless these two processes work together. Reading skills = decoding x language comprehension [15,16]. Problems related to the parts of the simple view model can cause difficulties related to reading skill development and reading comprehension. This is related to the processing resources such as inhibition, working memory and processing speed [17] (p. 86). In this respect, Borella et al. [18] (p. 541) have found that poor comprehenders do have problems related to specific inhibitory problems. Further studies by Borella and Ribaupierre [17] (p. 86) have indicated that working memory accounts for children’s performance in text comprehension. Freed et al. [19] (p. 135) pointed out that «vocabulary dominated other measures in explaining variance in comprehension». That means that the level of vocabulary is a robust tool to identify poor comprehenders. Studies have indicated that vocabulary knowledge is particularly modifiable to training [20].

## 3. Decoding

Children do not automatically learn the relationship between letters and sounds by seeing written language and recognizing word forms. Dehaene [21] points out in this respect that our brains are prewired for speaking and listening, but not for reading and writing. It is therefore of crucial importance to draw their attention to these con-nections and teach them the letters, their sounds and how they can be connected [22]. Dehaene and colleagues have shown [23] that when visual words are presented to adult readers, they systematically stimulate the visual word form area (VWFA), which is a specific region of the left-hemispheric ventral visual cortex [22] (p. 21). The VWFA «site is reproducible across individuals/scripts, attuned to reading-specific processes, and partially selective for written strings» [24] (p. 254). In this respect, Castles et al. [25] argue that it is important that children learn letters and their sounds, be able to connect two sounds, three sounds and finally break the reading code. Then, the child can read words and short sentences with capital and small letters.

## 4. Language Comprehension

Language comprehension is understanding and knowledge of spoken language and its structure [25]. Language comprehension is the basis of reading comprehension. Skilled readers have good language skills and a rich vocabulary, and therefore find it easy to understand the meaning of the text. Training on these elements is important, but when decoding is achieved, you must read a lot of text and focus on understanding the text you read [26]. Access to diverse texts that suit children’s different reading skills must therefore be good [1,2,3]. A person who does not have the power to decode cannot read. A person who cannot understand a text is not literate. To achieve good reading comprehension, students need to understand most of the words they read. Their language understanding becomes more important as the students get older and its roots lie in the vocabulary of each individual.

## 5. Challenges in Relation to Skill

**Figure 1:**
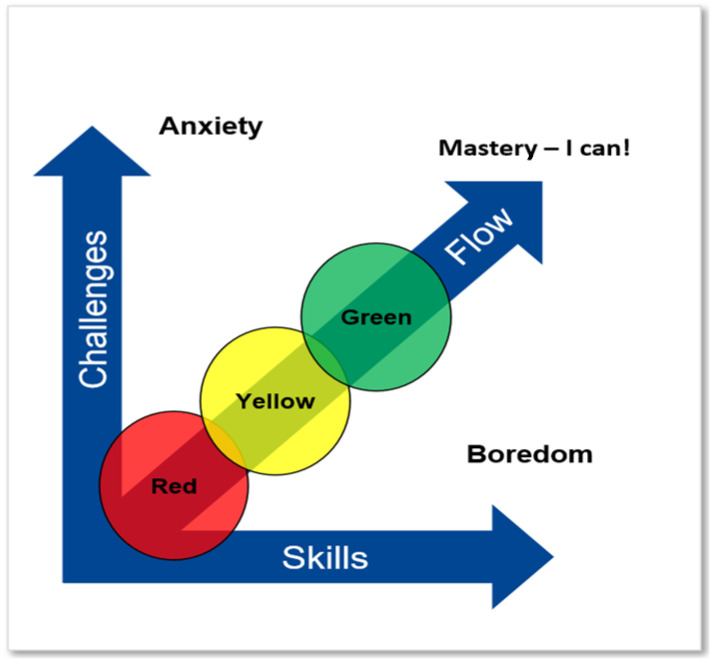
READ approach. The relation between challenges and skills in the READ approach. From the simple to the complex.

Csikszentmihalyi’s theory of flow (see Figure 1) is one of the key theories of the ‘Ignition’ project. As we can see in the figure, it is very important to go from the simple to the complex, where the challenges are in accordance with the skills. The key is that each individual receives the right challenges, which we call individual-oriented learning. This is how an individual gets into flow and acquires mastery [4,27]. Acquiring mastery and the feeling of ‘I CAN’ may increase the strong interest or passion for achievement in the individual [28,29]. The figure also indicates the different emphasis regarding the approach in the reading lesson. Phonics, systematic letter-sound knowledge, is taught in the beginning until the relevant individual has broken the reading code [26]. Then, the children receive training with an emphasis on improving vocabulary and reading comprehension.

The Ignition project (10-year follow-up longitudinal study) builds its approach to teaching reading to beginners on the ‘simple view of reading’ model [15]. It is supported by the world’s leading scholars in this field such as Heikki Lyytinen [30], Kate Nation [26], Maggie Snowling [31] and Stanislas Dehaene [22]. Research has shown that the model explains about 96% of reading skills [32]. In addition, the project is based on the theory of Csikszentmihalyi [4] and the theory of Ericsson on ‘deliberate practice’ [6].

## 6. Letter-Sound Knowledge

The “Letter-Sound knowledge” test [1] plays an important role in determining the position of each student, at the start of the school year (September), in middle of the school year (January) and in the end of the school year (May); it ensures that each student receives challenges based on his/her skills [4]. At the start of the school year (September) in the Vestmanna Islands, 58.3% of children in the first grade (*N* = 49, mean age: 5.76) could read words with capital letters or had broken the reading code, and 29.2% could read sentences with capital letters and 8.3% could read text. Based on these results, alphabet cards were sent home to practice connecting letters and sounds together with simple connection booklets. Great emphasis was placed on each student being challenged in reading based on their skills. This means, for example, that home reading books are suitable for every child and are neither too difficult for the child nor too easy. Reading books are marked according to difficulty levels, and when a child begins to read a simple text, he chooses books from the difficulty category which fits his skills. What skill level the child can choose from is guided by his or her teacher. As both Csikszentmihalyi [4], Ericsson and Pool [6] and Sigmundsson et al. [28,29,33] argue, the mentor/teacher/trainer has a key role in giving individuals the right challenges. Students in the first grade are divided across the year in so-called training hours where the teacher works with each group individually and gives the students appropriate training tasks. Those students who need training in letter and sound knowledge (group 1) received a special training session with a special teacher in a ‘study center’ where they worked with the basic aspects of literacy, phonological and phonemic awareness, and incorporated letters and their sounds are systematically practiced in every lecture. The children worked with sounds, rhymes, and syllables. The second group received similar training to the ‘study center’ group. There were more students in that group, but the guiding principle was always that challenges were based on skills. The third group also worked with phonological awareness, pairing upper and lowercase letters, finding the first sound in a word, practicing reading 3–4 letter words and working purposefully with listening comprehension [1]. The fourth group worked more with writing, writing single words and stories with their own spelling and working with word games. Those students also worked with the training material for increasing their vocabulary and reading longer words and text. In the fifth group, there were children who had started to read (are fully literate) and the tasks were weighted accordingly. The focus for these students was primarily on reading, reading comprehension and creative writing.

To evaluate the results, a reading framework was prepared in which the students’ reading and reading comprehension were assessed. To strengthen the teacher’s evaluation, four assessment tasks were presented. Students who did not break the reading code at the start of school and were at the first level of the reading framework took the letter-sound knowledge test again. On the next level of the framework, students read simple words and connected them to a picture. In the third part, it was checked whether the student was able to read short simple sentences and connect them to pictures (i.e., the student understands what he is reading). In the fourth stage, the last stage of the reading framework, students read a short text and answer questions from the text.

The results of the assessment of students in May indicated that 100% of the children had broken the reading code [3], which meant that they could read simple words with capital letters (September: 58.3% of the children were able to read simple words), 96% of the children could read sentences with capital letters (September: 29.2% of the children could) and 88% of the children could read text (September: 8% of the children could). In comparison with Norwegian children (school starters, average age 6.1 years), a study indicates that 11% had broken the reading code in September and 73% in May (3). When this article is being written, the results of the evaluation are being processed (October 2022, the second schoolyear to the child). The situation will be reassessed and the steps to be taken in the future will be decided. In other words, reassessment will include following ‘deliberate practice’: position assessment, follow-up, deliberate practice, position assessment, and follow-up [6].

## 7. READ Approach: Three Level

After the screening at the start of the school year (in September), we have information about the children’s level of reading skill.

In our approach, READ, we talk about three levels (see Figure 1):

RED (level 1): Emphasis on the teaching of letters and sounds and training in putting together the sounds of letters (two and two, three and three) so that it becomes simple words and short sentences. Goal: For the student to break the reading code. Assessment: The letter-sound test [1,2,3].

YELLOW (level 2): Emphasis on reading, training, and work with reading comprehension [26]. Work with simple texts and books with the right level of difficulty. In addition, emphasis is placed on writing and writing one’s own text. Goal: That students become fully literate. Assessment: FULLLÆS ‘fully literate’ assessment (a test that will be used at the end of the 2nd grade) is in progress and it is planned such that an assessment will be ready in the spring of 2023. Students in the first grade are not excluded from taking the fully literate position test if their skills have reached that level.

GREEN (level 3): Children are LITERACY (“fully literate”). The emphasis is on reading training and work with reading comprehension, writing and presentation (plays—play reading or reading with emphasis) [26]. Work is done with more difficult texts and books than at the YELLOW level. Goal: That students can fluently read different books and texts of their own choice. That students will also be able to write texts (creative writing). Emphasis is placed on text writing, reading comprehension and pronunciation.

Cooperation with the Vestmanna Islands Library is extremely important for the Kveikjum neistann! approach, where over 5000 children’s and adolescents’ books have been marked according to level of difficulty and field of interest. That marking is particularly handy, as you can suggest children and parents go to the library and pick up books at the right level of difficulty. This is in line with Csikszentmihalyi’s [4] perspective on flow. We should try to give children and youths challenges in line with their skills (action capacity), that gives better possibilities for mastery and self-efficacy [34].

## 8. Conclusions

Let’s promote reading skills and thus human resources. Build the practice on deliberate practice where there is a lot of training, repetition and follow-up which creates a neural network [9,10,11,12]. Parents/guardians and teachers are key people in that respect. The role of parents in their children’s education is extremely important. Children who grow up in homes where book reading is common, quickly develop an interest in the alphabet, even as early as the first three years of life [35]. Reading books increases vocabulary and reading comprehension, and the role of parents and other educators is by far the biggest influence on children’s vocabulary [25]. Read to your children, enjoy being together and use everyday situations to talk with your children. Visit the country’s well-stocked libraries. There is a great work going on where children and adults can find reading material that suits them.
